# Post-CaRMS match survey for fourth year medical students

**DOI:** 10.36834/cmej.69330

**Published:** 2020-07-15

**Authors:** Megan Clark, Sachin Shah, Lee Kolla, Stephanie Marshall, Sara Bryson, Bindu Nair

**Affiliations:** 1 University of Saskatchewan, Saskatchewan, Canada

## Abstract

**Background:**

We aimed to analyze which medical school experiences contribute to success in an increasingly competitive CaRMS match.

**Methods:**

We surveyed all matched University of Saskatchewan 2019 medical graduates on characteristics of their applications: number of program applications, interviews obtained, experiences (research, volunteer, leadership), awards and money spent on the residency match process, and qualitative reflections on the process. Using published CaRMS statistics based on number of positions versus applicants, specialties were divided into high availability/low demand (HA) (e.g. family and internal medicine) and low availability/high demand (LA) (e.g. dermatology and emergency medicine). Quantitative results were analyzed using descriptive statistics, chi-square and t-tests, and qualitative results thematically.

**Results:**

Data from 27 of 94 matched students were included. LA applicants were more likely to report at least one research project on their CV (66.67% among LA vs. 15.38% among HA, *n* = 27, χ2 = 8.640, *p* = 0.013), with a greater number of research presentations (mean=3.75 presentations vs. 2.07, *t* (25) = -2.251, *p* = 0.033). LA applicants had more elective weeks outside Saskatchewan (mean 11.75 weeks vs. 7.40 weeks, *t* (25) = -2.532, *p* = 0.018). Other application variables were not different between groups. Some students endorsed broader electives strategies, others (especially in surgical disciplines) supported narrower ones. Students reported travel, financial burden, document submission, and uncertainty as the greatest match process stressors.

**Conclusions:**

LA applicants cited more research projects and presentations, spent more elective weeks outside Saskatchewan, but were otherwise similar to HA applicants. Further studies should be done on student factors in the residency match process.

## Abstract (fr)

**Objectif:**

Nous visions à analyser quelles expériences durant le course de médecine contribuent à la réussite d’un jumelage de plus en plus compétitif par le CaRMS.

**Méthodes:**

Nous avons enquêté à propos des caractéristiques des candidatures de tous les diplômés en médecine jumelés en 2019 à l’Université de la Saskatchewan: nombre de demandes effectuées, les entrevues obtenues, les expériences (recherche, bénévolat, leadership), les prix et les sommes dépensées sur le CaRMS, et leurs réflexions qualitatives sur le processus du CaRMS. À l’aide des statistiques publiées par le CaRMS fondées sur le nombre de postes par rapport au nombre de candidats, les spécialités ont été réparties en haute disponibilité/faible demande (HA) (p. ex., médecine familiale et interne) et faible disponibilité/demande élevée (LA) (p. ex., dermatologie et médecine d’urgence). Les résultats quantitatifs ont été analysés à l’aide de statistiques descriptives, chi carré et tests t, et les résultats qualitatifs ont fait l’objet d’une analyse thématique.

**Résultats:**

Les données de 27 des 94 étudiants appariés ont été incluses. Les candidats LA étaient plus susceptibles de déclarer au moins un projet de recherche sur leur CV (66,67 % parmi les LA c. 15,38 % chez les HA, *n* = 7, χ2 = 8,640, *p* = 0,013), avec un nombre supérieur de présentations de recherche (moyenne = 3,75 présentations c. 2,07, *t* (5) = -2,251, p = 0,033). Les candidats LA avaient davantage de semaines de stages à option à l’extérieur de la Saskatchewan (moyenne 11,75 semaines c. 7,4 semaines, *t* (25) = -2,532, *p* = 0,018) Les autres variables des candidatures ne différaient pas entre les groupes. Certains étudiants souscrivaient à des stratégies de stages à option plus élargies, d’autres (plus particulièrement dans les disciplines chirurgicales) soutenaient des stratégies plus ciblées. Les étudiants ont signalé que les déplacements, le fardeau financier, la soumission de documents et l’incertitude comme les plus importants facteurs de stress du processus du CaRMS.

**Conclusions:**

Les candidats LA ont cité davantage de projets et de présentations de recherche, davantage de semaines de stages à option à l’extérieur de la Saskatchewan et plus de dépenses encourues en lien avec les stages à option, mais étaient autrement semblables aux candidats HA. D’autres études devront être réalisées sur les facteurs étudiants dans le jumelage du CaRMS.

## Introduction

At the University of Saskatchewan College of Medicine, the Office of Career Advising and Mentorship is available to students to help navigate the residency application process. One goal of this group is to objectively assess students’ application strategies and offer suggestions to make their application as competitive as possible. This includes discussing elective plans, interview practice, and Canadian Resident Matching Service (CaRMS) application review. Unfortunately, there is little objective evidence upon which to formulate plans and suggestions; many medical students feel the data they receive is limited to unsound anecdotal evidence. What data are available are often discipline specific. For example, Canadian medical students applying to Otolaryngology are six times more likely to match if they have at least one research paper compared to those who have none.^1^ Whether this relationship holds true for all specialties is unknown. Indeed, the Canadian Federation of Medical Students (CFMS) encourages “the provision of objective and easily accessible data describing the characteristics of those students who successfully matched to each residency program.”^2^ A paucity of data in this area continues to exist.

Graduating Canadian medical students apply for residency positions using CaRMS, a centralized application system and residency position match algorithm. Components of the CaRMS Curriculum Vitae include volunteer and leadership experience, awards, elective experiences, research, and other extracurricular activities. In past years, the residency match has become increasingly competitive with declining position to applicant ratio, with a nadir in 2018. This caused general public concern as well as concerted efforts by the Association of Faculties of Medicine of Canada (AFMC) and the CFMS to improve outcomes for medical student applicants.

Against this background, the purpose of this study was to investigate whether there were any differences in application content and strategy of students who successfully matched to high-availability/low-demand specialties (HA) versus those who successfully matched to low-availability/high-demand (LA) specialties, as well as to query participants’ subjective reflections on their lived experience through their match process. Both quantitative data and an understanding of matching students’ subjective concerns would better enable our office to support students through the process.

## Methods

To identify what strategies successful students employed to secure a residency position, we conducted an online survey of all 2019 University of Saskatchewan College of Medicine graduates who were successful in the match process. We stratified students’ responses based on their first-choice specialty. Specifically, as per published CaRMS 2019 data, students were stratified into high availability/low demand specialties (HA) and low availability/high demand specialties. High availability/low demand specialties (HA), such as family medicine and radiation oncology, have more available residency positions than applicants. Low availability/high demand specialties (LA), such as emergency medicine and dermatology, have more interested applicants than available positions.

Survey questions were built around factors potentially affecting their CaRMS match, based on the CaRMS online Curriculum Vitae (CV) components and the American National Residency Matching Service post-match survey.^8^ We asked graduates to indicate: the number of publications, presentations, awards, and research, leadership and volunteer experiences listed on their CV; their total number of applications submitted, interviews offered, interviews accepted and programs listed on their rank order list; and how much they spent on the CaRMS process and on electives (choosing from broad categories of cost ranges). We also asked applicants for their own personal reflections on the challenges of the CaRMS process, electives advice, application strategy, interview tips, and how well our College was supporting them through the process (survey included as Appendix A).

Results were analyzed using descriptive statistics, then we compared HA and LA groups using *t*-tests and *chi*-square tests as appropriate to the data. Qualitative results were analyzed by two authors using thematic analysis.^6^

This project received an ethics exemption as “program evaluation” by the University of Saskatchewan Behavioural Research Ethics Board (Beh ID 683).

## Results

Thirty-five students participated out of the 94 invited, for a response rate of 37%. Only 30 of those 35 students gave consent to publish their data in aggregate form, giving a reported response rate of 32%. Twenty-seven of those participants matched in the first iteration of the CaRMS R1 Main Residency Match in 2019. Due to their small group size, we excluded the data of the three participants who did not match in the first iteration of the CaRMS match. Students applying to HA (*n* = 15) and LA (*n* = 12) disciplines as their first-choice disciplines reported applying to a similar number of programs in preferred and other disciplines, receiving a similar number of interviews, and submitting a similar number of programs on their rank order lists (Table 1). Twelve students applied to more than one discipline (with three applying for HA as preferred discipline and nine to LA), but due to the small sample size, we analyzed participants’ data only by their first-choice specialty.

**Table 1:** Applications, interviews and programs ranked by LA and HA applicants.

Variable	HA mea *n, n*	LA mean, *n*	Statistical tests
Total program applications	17.3 (SD 11.5), *n* = 15	16.3 (SD 3.9), *n =* 12	t (25) = 0.292, *p* = 0.773
Preferred discipline program applications	16.3 (SD 12.0), *n* = 15	11.5 (SD 2.0), *n =* 12	t (25) = 1.347, *p* = 0.190
Interviews offered	11.0 (SD 5.3), *n* = 15	10.4 (SD 3.1), *n =* 12	t (25) = 0.334, *p* = 0.741
Number of programs ranked	13.60(SD 6.7), *n* = 15	14.2 (SD 4.1), *n =* 12	t (25) = -0.257, *p* = 0.799
Number of interviews accepted	9.7 (SD 4.9), *n* = 15	9.8 (SD 3.2), *n =* 12	t (25) = -0.101, *p* = 0.920
Research listed on CV (% of all applicants)	93.3%, *n* = 15	91.7%, *n =* 12	χ(1) = 0.027, *p* = 0.869
Publications listed on CV	1.6 (SD 3.0), *n* = 15	2.2 (SD 1.5), *n =* 12	χ(3) = 4.915, *p* = 0.178
Presentations listed on CV*	2.1 (SD 1.3), *n* = 15	3.8 (SD 2.5), *n =* 12	t (25) = -2.251, *p* =.033
Number of out-of-province elective weeks*	7.4 (SD 4.0), *n* = 15	11.8 (SD 4.9), *n =* 12	t (25) = -2.532, *p* =.018
Academic award (s) listed on CV (% of all applicants)	33.3%, *n =* 15	27.7%, *n =* 12	χ(2) = 1.406, *p* = 0.495
Number of academic awards, if awarded	2.2 (SD 1.6), *n =* 5	1.6 (SD 1.2), *n =* 3	t (6) = 0.487, *p* =0.643
Volunteer/leadership award listed on CV (% of all applicants)	28.6%, *n =* 14	16.6%, *n =* 12	χ(2) = 1.575, *p* = 0.455
Number of volunteer/leadership awards, if awarded	2.5 (SD 1.0), *n =* 4	2.5 (SD 0.7), *n =* 2	χ(2) = 1.575, *p* = 0.455
Number of student groups listed	3.2 (SD 2.4), *n =* 14	2.8 (SD 2.5), *n =* 12	*t* (24) = 0.484, *p* = 0.633
Number of student groups with leadership positions	2.1 (SD 2.5), *n =* 14	1.6 (SD 1.4), *n =* 12	*t* (24) = 0.596, *p* = 0.566
Number of volunteer activities listed	3.9 (SD 2.9), *n =* 15	4.4 (SD 3.1), *n =* 12	*t* (25) = -0.413, *p* = 0.683
Number of volunteer activities with leadership positions	2.2 (SD 2.6), *n =* 15	1.4 (SD 1.7), *n =* 12	*t* (25) = 0.903, *p* = 0.375

* indicates statistically significant difference

LA applicants were more likely to have research on their CaRMS CV in the discipline to which they matched: 66.67% of LA applicants vs. 15.38% of HA applicants, (*n* = 27, χ^2^ = 8.640, *p* = 0.013). There as a statistically significant difference between the number of presentations with a mean 2.0 presentations for HA applicants versus 3.8 for LA (*t* (25) = -2.251, *p* = 0.033). The difference in total number of publications listed on the CV was not statistically significant. LA applicants also spent more elective weeks outside Saskatchewan, at a mean of 11.8 weeks versus 7.4 weeks among HA applicants (*t* (25) = -2.532, *p* = 0.018). There were no other significant differences found between HA and LA applicants in any of the other components of the CaRMS CV (Table 1).

**Figure 1:**
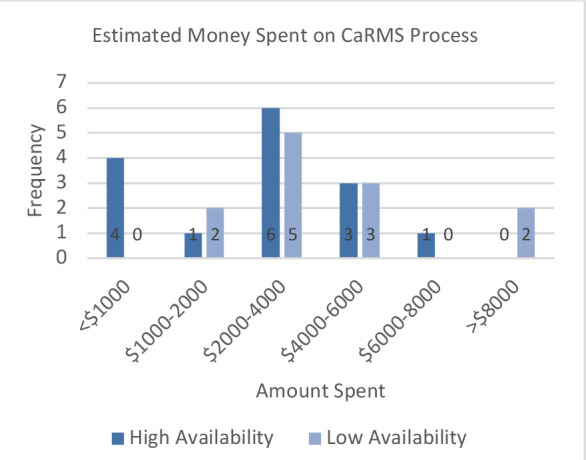
Estimated money spent on match process. *Participants were asked to estimate, in these broad categories, how much money they spent on the match process, including application fees and travel. Results did not differ between HA and LA groups (χ(5) = 9.923, p = 0.077)*.

**Figure 2:**
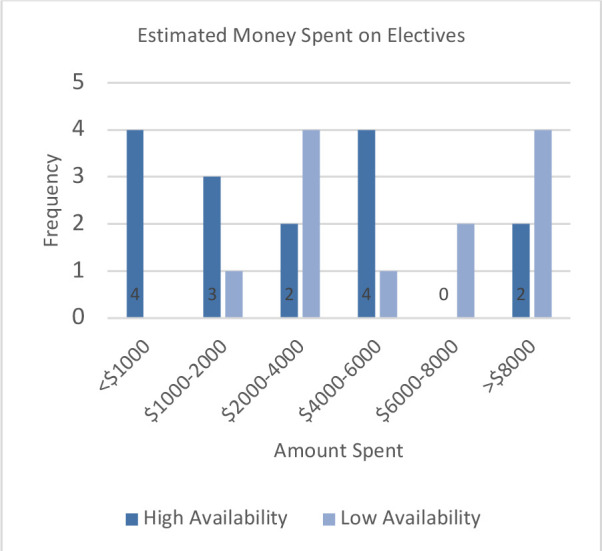
Estimated money spent on electives. *Students were asked to estimate, in these broad categories, how much money they spent on travel and application fees for electives. Results did not differ between HA and LA groups (χ(5) = 9.923, p = 0.077)*.

The amount of money students estimated they spent on the residency match process (Figure 1) did not differ between groups (χ(5) = 9.923, *p* = 0.077), nor did the amount of money spent on electives (χ(5) = 9.923, *p* = 0.077, Figure 2)).

Overall, 85.19% of matched participants who consented to having their data shared (*n* = 27) reported feeling adequately prepared for the residency match process. Some participants reported wishing they had completed more diverse electives in a broader range of specialties. Some participants who applied to LA surgical specialties reported wishing they had invested more elective time in their first-choice disciplines, with less time in lower-choice disciplines. One of these applicants who preferred a competitive surgical discipline stated, “I wouldn’t bother with backing up, I think it made my application to both specialties exponentially weaker.”

When asked what the most challenging part of the process was, travel and financial costs were most frequently reported. One participant shared, “The process of travel (away time and cost) adds significantly to the emotional burden of CaRMS. Being away from friends and family for extended periods of time accentuates the isolation and struggle to integrate into a new system/environment.” The application documents and interviews were also frequently reported as difficult. A few participants also reported the lack of transparency behind programs’ selection processes to be the most challenging part of the process, with two applicants calling the processes “random.”

Participants gave varying electives advice. Some participants promoted a broader, more diverse electives plan to strengthen applications to lower-choice disciplines and to create a more well-rounded clinical experience prior to starting residency. Others advocated for a narrower electives strategy, maximizing electives in the preferred discipline and at preferred sites. Many proponents of the latter strategy were applying to competitive surgical disciplines. A few participants discussed the difficulty of obtaining electives. Some suggested future medical students apply to multiple electives for each time block to have a backup plan should the preferred elective request be rejected.

Participants also gave varying advice on whether residency application strategies should be broad or narrow. Most participants advised to keep the strategy broad. A few participants, however, advised to keep the application strategy narrow, with one participant saying, “I think it went better going all in on what I wanted. With things getting more competitive it’s becoming more necessary to show your commitment to get your top choice.” When asked for interview advice, the most common guidance participants shared was to practice. The second most common was to put forward a confident, genuine, and mindful presence.

## Discussion

We examined the characteristics of successful applicants applying to both HA and LA specialties. Successful LA students had statistically significantly more participation in discipline specific research and weeks of out of province electives compared to HA students, likely because some LA residencies (plastic surgery, urology, and otolaryngology-head and neck surgery) are not offered in Saskatchewan and LA applicants choose to visit out-of-province programs to maximize exposure to their preferred discipline. These findings supported the commonly held belief that students pursuing a LA specialty should maximize their exposure and appearance of commitment to their desired field. However, all other factors commonly believed to improve one’s success in the match process, like awards and volunteer/leadership experience, did not appear to differ between groups.

Most respondents felt a broad elective strategy was most advantageous. A minority of responders recommended a narrow, discipline specific elective strategy; these were most commonly students pursuing a LA surgical specialty. Given the new AFMC student electives diversification policy of eight weeks maximum per direct entry discipline,^7^ students applying for a LA specialty may feel their application is weakened by this limited exposure. Research does not support this claim, and these students may have been biased toward that strategy by matching to their preferred LA discipline. Interestingly, despite the narrow elective strategy advocated by applicants to LA specialties, previous studies suggest a less narrow strategy is equally efficacious, even for LA specialties.^3^

A common theme emerged among respondents when asked about the most difficult aspects of the elective/application process; many felt travel and the financial cost of electives and the residency match process (applications, flights, hotels, etc.) created the most stress, both financial and emotional. Preparing match documents and the interview process were also commonly reported by students as challenging. A few participants called the match process seemingly “random”, which we interpret as arbitrary, with applicants not understanding the inner workings of programs’ selection processes.

Although all students experience to some degree the inherent anxiety of navigating through this process, future support and education could help mitigate some of this stress: cost estimates of the process, financial support, and continued elective planning/application review and interview support for students. Our study has echoed previous findings suggesting that the residency match process experience is stressful.^4^ To help navigate this some centers have adopted peer-to-peer or near-peer information sessions. These have been viewed favorably by students^5^ and our program has begun implementing them.

## Limitations

Our study’s generalisability is limited by its small sample size from one year at one medical school, with a reported response rate of only 32%. Self-reporting bias could also have affected results, especially given our Office’s counselling relationship with students. Due to its small sample size, our study is also limited by its lack of information on strategies employed by unmatched applicants (*n* = 3, which were excluded from analysis).

## Conclusion

Overall, no strong recommendations can be drawn from these data, and further research is needed to help students navigate this aspect of the elective/application process. Our paper emphasizes that successful applicants applying to HA and LA specialties employ similar application strategies. The residency match process, as a whole, remains a stressful experience for applicants. Increasing career advising supports for applicants throughout the process, integrating participants’ lived experience with objective data, may help alleviate some of the stress experienced.

## Appendix A: Survey

1. May we include your data in any reports for internal use or external publication? [Yes/No]
2. Did you match in CaRMS 2018? [Yes, in the first Iteration/ Yes, u=in the second Iteration/No/Prefer not to say]
3. Which specialty did you match to? [drop-down list of specialties from CaRMS website (see bottom of document for list), plus None” and “Prefer not to say”]
4. Which specialty was your #1 choice? [drop-down list of specialties from CaRMS website, plus “None” and “Prefer not to say”]
5. I matched to my specialty choice #: [options #1-12, plus “Prefer not to say”]
6. Matched to program choice: [options #1-12, Greater than 12, plus “Prefer not to say”]
7. Number of Applications
  - How many applications did you submit to all programs in all disciplines? This includes programs with multiple sites if separate application submissions through the CaRMS portal were involved (Ex. Usask-family medicine-Regina and Usask-family medicine-Prince Albert). [options any ordinal value 1-100, plus “Prefer not to say”]
  - How many applications did you submit to programs in your 1st choice specialty? [options any ordinal value 1-100, plus “Prefer not to say”]
  - How many applications did you submit to programs in your 2nd and/or lower choice specialties? [options any ordinal value 1-100, plus “Prefer not to say”]
8. Number of Interviews Offered
  - How many interviews were you offered in total, across all disciplines? [options any ordinal value 1-100, plus “Prefer not to say”]
  - How many interviews were you offered in your 1st choice specialty? [options any ordinal value 1-100, plus “Prefer not to say”]
  - How many interviews were you offered in your 2nd and/or lower choice specialties? [options any ordinal value 1-100, plus “Prefer not to say”]
9. Number of Interviews Accepted
  - How many interview offers did you accept in total, across all disciplines? [options any ordinal value 1-100, plus “Prefer not to say”]
  - How many interview offers did you accept in your 1st choice specialty? [options any ordinal value 1-100, plus “Prefer not to say”]
  - How many interview offers did you accept in your 2nd and/or lower choice specialties? [options any ordinal value 1-100, plus “Prefer not to say”]
10. Number of Programs Ranked
  - How many programs, across all disciplines, did you submit on your rank order list? [options any ordinal value 1-100, plus “Prefer not to say”]
  - How many programs in your 1st choice specialty did you submit on your rank order list? [options any ordinal value 1-100, plus “Prefer not to say”]
  - How many programs in your 2nd or lower choice specialty did you submit on your rank order list? [options any ordinal value 1-100, plus “Prefer not to say”]
11. Did you list research experience on your CaRMS CV? [Yes/No/Prefer not to say]
  - Was the research in the discipline you matched to? [Yes/No/Not exactly but related/Prefer not to say]
  - Was the research in your 1st choice discipline? [Yes/No/Not exactly but related/Prefer not to say]
12. How many publications did you list on your CaRMS CV? [options any ordinal value 0-50, plus “Prefer not to say”]
  - Were these publications in the discipline you matched to? [Yes/No/Some publications were and some weren’t/Not exactly but related/Prefer not to say]
  - Were these publications in your 1st choice discipline? [Yes/No/Some publications were and some weren’t/Not exactly but related/Prefer not to say]
13. How many presentations did you list on your CaRMS CV? [options any ordinal value 0-50, plus “Prefer not to say”]
  - Were these presentations in the discipline you matched to? [Yes/No/Some presentations were and some weren’t/Not exactly but related/Prefer not to say]
  - Were these presentations in your 1st choice discipline? [Yes/No/Some presentations were and some weren’t/Not exactly but related/Prefer not to say]
14. Electives
  - How many weeks of electives did you do in your matched discipline? [options any ordinal value 0-30, plus “Prefer not to say”]
  - How many weeks of electives did you do at the program you matched to? [options any ordinal value 0-30, plus “Prefer not to say”]
  - How many weeks did you do in other disciplines? [options any ordinal value 1-30, plus “prefer not to say”]
  - How many weeks of electives did you do outside Saskatchewan? [options any ordinal value 1-30, plus “prefer not to say”]
  - How many weeks of electives did you do in each of the below specialties?
    (Select a CaRMS direct-entry specialty below and then select the applicable number of weeks)
  - § Number of weeks in other disciplines: see list of CaRMS direct-entry specialties below: students to select specialty name then fill in ordinal number of weeks
15. Did you have a note that you were in the top quartile of your class academically on your MSPR? [Yes/No/Not applicable/Prefer not to say]
16. Did you have any professionalism issues noted on your MSPR? [Yes/No/Prefer not to say]
17. In which year did you start medical school? [years 2010-2016 listed, plus “Prefer not to say”]
18. How much money do you estimate you spent on the CaRMS process, between carms.ca, travel for CaRMS interviews, and other expenses? [select 1 option: <$1000, $1000-2000, $2000-4000, $4000-6000, $6000-8000, >$8000, Prefer not to say]
19. How much money do you estimate you spent on electives between application fees, travel, accommodations, and any other expense? [select 1 option: <$1000, $1000-2000, $2000-4000, $4000-6000, $6000-8000, >$8000, Prefer not to say]
20. Were there any academic concerns noted on your MSPR? [Yes/No/Prefer not to say]
21. How many clerkship rotations did you have to remediate? [selectable ordinal number 0-20, Prefer not to say]
22. Did you receive any academic awards during medical school that you listed on your CaRMS CV? [Yes/No/Prefer not to say]
  - If yes, how many? [ordinal # 1-20, Prefer not to say]
23. Did you receive any volunteer or leadership awards during medical school that you listed on your CaRMS CV? [Yes/No/Prefer not to say]
  - If so, how many? [ordinal # 1-20, Prefer not to say]
24. How many student groups did you list on your CaRMS CV (such as the SMSS or a specialty interest group)? [ordinal # 0-20, Prefer not to say]
  - How many of these groups did you list leadership (executive position) in on your CaRMS CV? [ordinal # 0-20, Prefer not to say]
25. How many volunteer activities did you list on your CaRMS CV? [ordinal # 0-20, Prefer not to say]
  - How many of these volunteer activities did you list as having a leadership position in (Ex. volunteer coordinator, sat on board, organizer)? [ordinal # 0-20, prefer not to say]
26. Did you feel adequately prepared for the CaRMS match? [Yes/No/Prefer not to say]
27. If doing it all over again, would you make the same decisions in regards to electives, leadership/volunteer activities, research, and program applications? [Yes/No/Prefer not to say]
  - If no, what would you change? [open-form comments box, with option to leave blank]
28. What was the most challenging part of CaRMS? [open-form comments box, with option to leave blank]
29. Any reflections you’d like to share on how you distributed your electives? [open-form comments box, with option to leave blank]
30. Any reflections you would like to share on your general application strategy? [open-form comments box, with option to leave blank]
31. Any tips to share for CaRMS interviews? [open-form comments box, with option to leave blank]
32. How is the U of S doing in supporting you in the CaRMS match? [ordinal boxes with Likert scale: 1=not well, 2=somewhat, 3=moderately well, 4=quite well, 5=very well; plus open-form comments box, with option to leave blank]
33. How could the U of S better support you in the CaRMS match? [open-form comments box, with option to leave blank]
34. Anything else you’d like to share? [open-form comments box, with option to leave blank]
  List of CaRMS direct-entry specialties
  Anatomical Pathology
  Anesthesiology
  Cardiac Surgery
  Dermatology
  Diagnostic Radiology
  Emergency Medicine
  Family Medicine
  General Surgery
  Hematological Pathology
  Internal Medicine
  Medical Genetics and Genomics
  Medical Microbiology
  Neurology
  Neurology - Pediatric
  Neuropathology
  Neurosurgery
  Nuclear Medicine
  Obstetrics and Gynecology
  Ophthalmology
  Orthopedic Surgery
  Otolaryngology - Head and Neck Surgery
  Pediatrics
  Physical Medicine & Rehabilitation
  Plastic Surgery
  Psychiatry
  Public Health and Preventive Medicine
  Radiation Oncology
  Urology
  Vascular Surgery
